# The Cerebro-Spinal Fluid; Its Spontaneous Escape from the Nose

**Published:** 1900-06

**Authors:** 


**The Cerebro-Spinal Fluid; its Spontaneous Escape from the Nose.** By StClair Thomson, M.D. Pp. vii., 140. London: Cassell & Company, Limited. 1899.

For many years after the time of Galen it was believed that the fluid in the ventricles of the brain found a regular exit from the nose by way of the infundibulum. Schneider, in 1655, was the first to show that the fluid discharges from the nose came from the mucous membrane, and that there was no direct communication between the nose and the ventricles of the brain. Galen, however, was not so much in error as is generally supposed, for Dr. StClair Thomson has met with the case of a young girl who suffered from a constant dripping of fluid from the nostrils, amounting to about fifteen ounces in twenty-four hours, and this fluid when examined was found to be cerebro-spinal fluid. It caused her no inconvenience beyond the annoyance of a constant dripping, and all efforts at alleviating the condition proved futile. It was evidently an abnormal physiological condition due to an open canal between the cranial fossa and the olfactory cleft, and its interest depended chiefly upon its rarity. The author has collected notes of nine other cases where the discharge from the nose was undoubtedly cerebro-spinal fluid, and of numerous others where it was so in all probability, so that the condition must be looked upon as one which may be met with from time to time.

Dr. Thomson took the opportunity to make some interesting observations upon the composition and function of the cerebro-spinal fluid in the human subject, in which investigation he was assisted by Professor Halliburton, and he gives us the results of his labours in an interesting form in the present volume.

